# Preschool hyperactivity specifically elevates long-term mental health risks more strongly in males than females: a prospective longitudinal study through to young adulthood

**DOI:** 10.1007/s00787-016-0876-8

**Published:** 2016-06-13

**Authors:** Elizabeth Smith, Brenda J. Meyer, Johanna Koerting, Cathy Laver-Bradbury, Louise Lee, Harriet Jefferson, Kapil Sayal, Luke Treglown, Margaret Thompson, Edmund J. S. Sonuga-Barke

**Affiliations:** 1 Department of Psychology, University of Bath, Bath, UK; 2 Developmental Brain and Behaviour Laboratory, Psychology, University of Southampton, Southampton, SO17 1BJ UK; 3 CAMHS, Better Care Centre, Solent NHS Trust, Southampton, UK; 4 Division of Psychiatry and Applied Psychology, School of Medicine, University of Nottingham, Nottingham, UK; 5 Centre for ADHD and NeuroDevelopmental Disorders Across the Lifespan (CANDAL), Institute of Mental Health, University of Nottingham, Nottingham, UK; 6 Department of Psychology, University College London, London, UK

**Keywords:** Preschool hyperactivity, Long-term risk, Mental health, Longitudinal study

## Abstract

Evidence of continuities between preschool hyperactivity and adult mental health problems highlights the potential value of targeting early identification and intervention strategies. However, specific risk factors are currently unclear. This large-scale prospective longitudinal study aimed to identify which hyperactive preschoolers are at the greatest long-term risk of poor mental health. One hundred and seventy children (89 females) rated as hyperactive by their parents, and 88 non-hyperactive controls (48 females) were identified from a community sample of 4215 3-year-olds. Baseline data relating to behavioral/emotional problems and background characteristics were collected. Follow-up mental health and functional impairment outcomes were collected between 14 and 25 years of age. At age 3 years, males and females in the hyperactive group had similarly raised levels of hyperactivity and other behavior problems. In adolescence/young adulthood, these individuals showed elevated symptoms of ADHD, conduct disorder, mood disorder, anxiety and autism, as well as functional impairment. Preschool hyperactivity was strongly predictive of poor adolescent/adult outcomes for males across domains with effects being specifically driven by hyperactivity. For females, the effects of preschool hyperactivity were smaller and dropped to non-significant levels when other preschool problems were taken into account. Environmental risk factors also differed between the sexes, although these may also have been mediated by genetic risk. In conclusion, these results demonstrate marked sex differences in preschool predictors of later adolescent/adult mental health problems. Future research should include a measure of preschool inattention as well as hyperactivity. The findings highlight the potential value of tailored approaches to early identification strategies.

## Introduction

Prospective longitudinal studies confirm that the developmental processes that determine adult mental health have their roots in early childhood [1, 2]. There is now compelling evidence that early, premorbid, behavioral markers of long-term risk for mental health problems are present in the preschool years—even in children as young as 15-month-olds [3]. Preschool hyperactivity, and its correlated elements of impulsivity and inattention, has been shown to be associated with academic underachievement [4, 5] and mental health disorders in late adolescence [5, 6]; and anti-social activities and drug use in adulthood [7–9]. It is also associated with substantially increased service burden from childhood onward [10]. Furthermore, it appears that hyperactivity itself, at least in part, drives such associations rather than other co-occurring behavioral problems [4]. The progression from childhood hyperactivity into these long-term negative outcomes is complex and may incorporate different developmental risk pathways [11, 12]. Both homotypic and heterotypic continuities exist [13]. For example, Lahey et al. found that meeting ADHD criteria in preschool was highly predictive of continued ADHD symptoms and functional impairment at school age [14], while Bufferd et al. demonstrated that preschool hyperactivity in children aged 3 years predicted a diagnosis of oppositional defiant disorder (ODD) at age 6 years [13]. Preschool hyperactivity has also been associated with later emotional problems and poor social skills [6].

These identified continuities highlight the possible value of interventions targeted at preschool hyperactivity to reduce the long-term risks of mental health conditions [5, 15–17]. However, the costs of behavioral parent training, the first-line recommended treatment for preschool children with attentional/hyperactivity problems [18, 19], is potentially high [20]. It is, therefore, important to be able to identify which hyperactive children are at long-term risk of problems later in life to target those that would benefit most from preschool interventions. Currently, little is known about the specific features associated with preschool hyperactivity that place children at particular risk of poor long-term outcomes. We, therefore, aimed to address this gap in understanding of the associations between preschool hyperactivity and late adolescence/early adult mental health outcomes in a prospective longitudinal study.

A number of factors may be important in this regard. First, risk associated with preschool hyperactivity may vary as a function of sex [21–26]. ADHD is more common in males than females [27–29] with a ratio of between 16:1 and 3:1 reported in clinical samples [27] and between 3.2:1 and 1.9:1 in population samples [29]. A number of theories have been proposed to explain these differences [21, 23, 27, 30–34]. Yet, initial risk behaviors (i.e., hyperactivity) are present in early development in both males and females. For example, ADHD sex ratios derived from non-referred samples in the preschool period are more balanced than in later childhood. Ratios have been estimated at between 1.6:1 and 1.8:1 in children aged 3–5 years [29, 35]. Of interest, in a 6-year longitudinal study of hyperactive preschool children, parent-reported hyperactivity and impulsivity ratings were higher for females versus males at baseline, but showed greater decline in symptoms over time [16]. These results suggest that growing up female and hyperactive, in some way, is associated with reduced risk of a poor outcome compared with growing up male and hyperactive. Rutter et al. identified three levels of potential causal mechanisms for sex differences in psychopathological conditions: genetic influences (e.g., genetic expression of phenotype); hormonal/maturational consequences (e.g., environmental/biological exposure); and proximal risk factors (e.g., different protectives vs. vulnerability mechanisms) [36]. There is some evidence to suggest that females with ADHD develop better coping strategies than males [21], or elicit different parental responses [37]. In contrast, Lahey et al. found that females who showed preschool hyperactivity exhibited more anxiety and depression during adolescence than did their comparison peers without ADHD. Furthermore, these increases were significantly greater than those seen in males with preschool hyperactivity during the same period [30]. Other studies have shown a greater likelihood of internalizing behavior in females versus males with ADHD [21, 22, 25, 26, 30], and a greater likelihood of externalizing behavior in males versus females [21, 24–26]. Given the evidence of a different trajectory of hyperactivity and associated symptoms in males and females across the lifespan, preschool risk factors may also differ according to sex, and justify further exploration.

Second, the presence of co-occurring emotional and conduct problems might also carry an additional long-term risk. Emotional and behavioral problems displayed by hyperactive and non-hyperactive individuals alike have been shown to predict internalizing and externalizing problems in preadolescence [16, 38–42].

Third, developmental delay (DD) may be important. In a comparison of preschool children with and without DD, Baker et al. found that 54.5 % of those with DD had comorbid mental health disorders compared with 23.5 % of the typically developing children. Of these, 52.9 % met symptoms of ADHD and ODD, compared with 21.4 % of children without DD [43]. Furthermore, preschool children with DD are significantly more likely to develop later internalizing and externalizing problems than those without DD [44].

Finally, family background characteristics could exacerbate risk. Socioeconomic disadvantage has been linked to ADHD in two recent studies [45, 46]. Poor parental education has been shown to predict poor outcomes in children with preschool behavior problems [47], and family discord and dissolution have repeatedly been shown to be related to poor mental health outcomes [47–50].

The current study had three specific goals: (1) to compare the long-term risk of mental health problem in groups of hyperactive and non-hyperactive preschoolers selected from a large community sample and test whether these risks affect males and females differently; (2) to establish whether these effects are independent of other preschool behavioral characteristics; and (3) to identify factors that predict poor outcomes in the group of hyperactive preschoolers (i.e., which hyperactive preschoolers go on to have problems?).

## Methods

### Study design

A prospective cohort study was initiated between 1989 and 1997 within the New Forest and City of Southampton area, Hampshire (England). Baseline data were collected from medical records of children living in the area along with behavioral and demographic questionnaires administered during routine developmental health checks at age 3 years. Children with and without high levels of hyperactivity were identified by total scores on the parent-reported Werry–Weiss–Peters Activity Rating Scale (WWP) [51]. Follow-up data, assessing mental health outcomes and impairment, were collected from consenting participants between 2010 and 2014, when the ages of participants ranged from 14 to 25 years.

### Participants

#### Baseline

A total of 4215 children aged 3 years living in the Southampton area were included in developmental checks conducted by family health visitors within the prespecified time frame. Of these, 543 children had high levels of hyperactivity (top 17.2 % of parent reported scores of ≥20 on the WWP) and had basic demographic information available.

#### Follow-up

Between 2010 and 2014, when their ages ranged from 14 and 25 years, 499 (204 female) of the 543 eligible participants were traced and recontacted. Follow-up data were collected from 170 (34.1 %; 89 females). These were the hyperactive group. Of the remainder, 87 declined to take part, 240 were non-responders, and two were deceased. A further 299 children were selected at random from the sample of those who did not meet the hyperactive symptom threshold at age 3 years (WWP <20), and 189 were traced and recontacted. Of these, 88 (46.6 %; 48 female) agreed to take part. These were the control group. Of the remainder, 26 declined and 75 did not respond.

To check the representativeness of the contributing sample, we compared hyperactive and control participants with their non-participating counterparts on variables collected at baseline when they were aged 3 years (Table 1). Groups did not differ as a function of participation except that male control participants had significantly lower hyperactivity scores but lived in more deprived neighborhoods than male control non-participants. The difference for hyperactivity became non-significant (*p* > 0.15) when cases, which could not be traced (current addresses not obtained), were excluded. This suggests that the differences between male control participants and non-participants were mainly due to a difficulty obtaining contact addresses rather than to active decisions not to participate.

**Table 1:** Baseline comparisons on key measures (aged 3 years) between follow-up participating and non-participating males and females (hyperactive vs. control groups)

		Participants		Non-participants		Statistics	
		*n*	Mean (SD)	*n*	Mean (SD)	*t*	*p*
Hyperactive group							
Males	Hyperactivity	81	27.51 (6.74)	236	28.23 (7.65)	−0.76	0.45
	Conduct	77	2.95 (1.45)	218	3.33 (1.70)	−1.88	0.06
	Emotional	77	1.34 (1.13)	219	1.51 (1.28)	−1.04	0.30
	Deprivation	80	−1.06 (1.78)	201	−1.05 (1.96)	−0.04	0.97
Females	Hyperactivity	89	27.80 (7.95)	137	28.21 (7.68)	−0.39	0.70
	Conduct	82	2.71 (1.64)	126	2.79 (1.64)	−0.30	0.77
	Emotional	82	1.32 (1.22)	125	1.54 (1.44)	−1.13	0.26
	Deprivation	87	−0.92 (1.97)	113	−0.95 (1.78)	−0.12	0.91
Control group							
Males	Hyperactivity	40	7.90 (5.01)	118	10.66 (5.07)	−2.99	<0.01
	Conduct	40	1.43 (0.93)	118	1.81 (1.25)	−1.74	0.08
	Emotional	40	0.88 (1.07)	118	1.04 (1.12)	−0.83	0.41
	Deprivation	39	−0.85 (2.03)	116	0.15 (2.51)	−2.25	0.03
Females	Hyperactivity	48	8.43 (4.71)	92	9.51 (5.09)	−1.23	0.22
	Conduct	47	1.21 (1.10)	93	1.56 (1.09)	−1.77	0.08
	Emotional	47	0.80 (0.94)	93	0.94 (0.89)	−0.85	0.40
	Deprivation	48	−1.26 (2.38)	89	−0.41 (2.53)	0.63	0.53

Deprivation is based on the Carstairs index; Hyperactivity is based on the Werry–Weiss–Peters activity rating scale; conduct and emotional problems are based on the behavior checklist

### Procedure

The study received ethical approval from the University of Southampton and the National Health Service Research ethics committees. Parents of the participants provided written informed consent or gave verbal consent for future contact and participation in the study at the time of the 3-year developmental check. Participants and parents also provided written informed consent to provide follow-up data, once contact had been reestablished. The majority of follow-up data were collected via face-to-face interviews conducted either in the individuals’ homes, or in a research room at a clinic according to the preferences of the participants. Some control families (*n* = 38) completed the questionnaires online. Interviews lasted approximately 1 h. A sum of £20 was made to each participant to reimburse them for costs incurred. Birth and health history was also extracted from medical records with the permission of the participants and their parents, or via self-report where these records could not be obtained.

### Measures

#### Baseline (whole sample at age 3 years)

The following child demographic information was recorded: age, sex, and ethnicity. Parental demographic information was recorded as binary variables: parents’ relationship status (biological parents living together vs. living apart when child was aged 3 years) and parents’ level of education [high vs. low; where high education was defined as achieving qualifications above those taken in school at age 16 years (UK, GCSE level)].

Preschool hyperactivity was assessed using the WWP [51]—a 27-item scale measuring hyperactivity in young children. Examples of items on the scale include ‘During meals, is the child up and down at the table?’; ‘When watching television does the child talk too much?’; ‘When at play does the child disrupt the play of other children?’ Parents provide responses on a Likert scale (no/some/much/or N/A). The scale has good levels of reliability and validity [52], correlates with other measures of hyperactivity, and predicts levels of hyperactivity 5 years after initial testing [41].

Other preschool behavioral problems were recorded on the behavior checklist (BCL) [53]—a revised 19-item parent report questionnaire with good psychometric properties [54]. The subscales related to conduct (i.e., poor social adjustment), emotional problems (i.e., poor emotional adjustment), sleep, feeding, and soiling problems are reported here.

Economic deprivation was measured using the Carstairs index of deprivation [55], based on characteristic data regarding families living in different postcode regions in the UK in 1991. Scores from four factors of this index (unemployment, overcrowding, social class, and car access) were standardized to UK norms and converted into a total deprivation score with higher scores reflecting greater deprivation.

#### Baseline (for hyperactive group only)

Pregnancy and birth histories included premature birth (<37 weeks gestation), low birth weight (<2500 g), and complications during labor. These were obtained from medical records, or self-report where such records were unavailable, and recorded as binary categorical data.

Developmental delay (DD) was assessed via separate measures of (1) speech and language delay, and (2) cognitive delay were determined by health visitors using standardized coding terms [satisfactory, problem, observation, treatment, referred, not examined (SPOTRN)] within the personal child health record (PCHR) when each child was aged 3 years. The PCHR is a national standard health and development record used by health professionals, regularly reviewed by the Royal College of Paediatrics and Child Health [56].

#### Follow-up measures (14–25 years)

Adolescent/young adult psychopathology was assessed using the parent report version of the Conners Comprehensive Behavior Rating Scales (CBRS) [57] and was adapted (with the agreement of the publisher) to assess the mental health status of participants at follow-up. More specifically, a number of items were modified to make them developmentally relevant for the study sample. The CBRS has reliable psychometric properties, including good validity, internal consistency, inter-rater reliability, and test–retest reliability [57]. The data from the CBRS can be used to derive a range of different metrics. For the purpose of this study, the focus was on CBRS subscales for ADHD, conduct disorder (CD), oppositional defiant disorder (ODD), autism spectrum disorders (ASDs), mood disorders, and anxiety disorders. These outcomes do not reflect a clinical diagnosis, but reflect symptoms of severity at a level consistent with core categories from the diagnostic and statistical manual of mental disorders: fourth edition (DSM-IV-TR™) [58].

Impairment was measured using the Weiss Functional Impairment Rating Scale—self-report (WFIRS–S) [59], which evaluates everyday functioning across a range of settings and domains. It comprises six subsections: home (8 items); learning and work (12 items); activities of daily living (14 items); self-concept (4 items); social activities (6 items); and risky activities (13 items). It forms part of the Canadian ADHD Resource Alliance (CADDRA) toolkit and has been psychometrically validated for use in the ADHD population [60, 61].

### Data analysis

First, baseline characteristics were compared for hyperactive and control groups as a function of sex of child, using ANOVA. Second, homotypic and heterotypic continuities were examined using two-way multivariate analysis of variance (MANOVA) to examine levels of adolescent/young adult ADHD as a function of group (hyperactive vs. control) and sex, with symptoms of ADHD, conduct problems (ODD and CD), mood problems (depression and mania); anxiety (generalized anxiety, obsessive compulsive disorder, and social phobia); and ASD symptoms (Asperger’s syndrome and autism) as outcome variables. Impairment (total WFIRS–S score) was assessed in a separate univariate ANOVA. Where significant multivariate effects were observed in these analyses, simple main effects of group for each sex were assessed for each variable along with the univariate group by sex interaction. Third, to establish the independent contribution of preschool hyperactivity to long-term mental health, the BCL subscales (emotional, conduct, sleep, feeding, and soiling problems) were added as covariates to the above models. Finally, taking the hyperactive group alone, we conducted multiple regression analyses to examine which baseline factors predicted poor outcomes. One analysis included severity of hyperactivity, the BCL subscales and DD, a second examined prenatal and perinatal risk. A third examined the importance of demographic factors.

## Results

Demographic and baseline clinical characteristics of the hyperactive and control groups at age 3 years are presented in Table 2, split by sex of child. After adjusting for multiple testing, there was a significant effect of group on WWP hyperactivity, BCL conduct, emotion, sleep, and feeding problems. Those in the hyperactive group had higher scores, on average, than those in the control group (see Table 2). There were no effects of sex and no sex by group interactions. Male and female children in the hyperactive group were equally impaired across all outcomes. Hyperactive and control groups did not differ on demographic background factors.

**Table 2:** Comparison of baseline measures using two-way (group by sex of child) ANOVAs for continuous outputs and logistic regression for categorical outputs

	Male, % or mean (SD)		Female, % or mean (SD)		Statistics		
	Hyperactive	Control	Hyperactive	Control	Group	Sex	Interaction
Demographics	*n* ^a^ = 80	*n* ^a^ = 39	*n* ^a^ = 87	*n* ^a^ = 48			
Deprivation^a^	−1.06 (1.78)	−0.85 (2.03)	−0.92 (1.97)	−0.13 (2.38)	*F* = 3.57	*F* = 2.62	*F* = 1.20
Parent education	53.8 %	25.0 %	53.9 %	40.0 %	*ß* = −0.56	*ß* = 0.68	*ß* = −0.69
Parents living apart	9.9 %	0 %	15.9 %	7.1 %	*ß* = −0.90	*ß* = 17.66	*ß* = −18.15
Behavioral problems	*n* ^b^ = 77	*n* ^b^ = 40	*n* ^b^ = 82	*n* ^b^ = 47			
Conduct^b^	2.95 (1.45)	1.43 (0.93)	2.72 (1.66)	1.21 (1.10)	*F* = 65.59^**+**^	*F* = 1.45	*F* = 0.00
Emotion	1.34 (1.13)	0.88 (1.07)	1.32 (1.23)	0.80 (0.94)	*F* = 10.75^**+**^	*F* = 0.11	*F* = 0.04
Sleep	2.60 (1.18)	1.75 (1.50)	2.56 (1.95)	1.10 (0.99)	*F* = 26.50^**+**^	*F* = 2.36	*F* = 1.82
Toileting	0.76 (1.13)	0.41 (0.75)	0.42 (0.90)	0.23 (0.59)	*F* = 4.85	*F* = 4.79	*F* = 0.49
Feeding	2.66 (1.89)	2.08 (1.69)	2.49 (1.94)	1.32 (1.33)	*F* = 12.41^**+**^	*F* = 3.57	*F* = 1.41
Hyperactivity	27.51 (6.74)	7.90 (5.01)	27.80 (7.95)	8.43 (4.71)	*F* = 495.60^**+**^	*F* = 0.22	*F* = 0.02

Hyperactivity ratings are based on Werry–Weiss–Peters activity rating scale; conduct, emotional, sleep, toileting, and feeding problems are based on the behavior checklist. Sidak’s alpha (*p* < 0.05) level corrected for nine tests with a multicorrelation of 0.15 = *p* < 0.006)

^+^ Significant at *p* < 0.05 after correcting for multiple testing

^a^ *n*’s represent deprivation data only. ^b^ *n*’s represent conduct data only. *n*’s for other variables may differ slightly due to missing data

### Is preschool hyperactivity associated with poor outcomes in adolescence/adulthood?

**Fig. 1:**
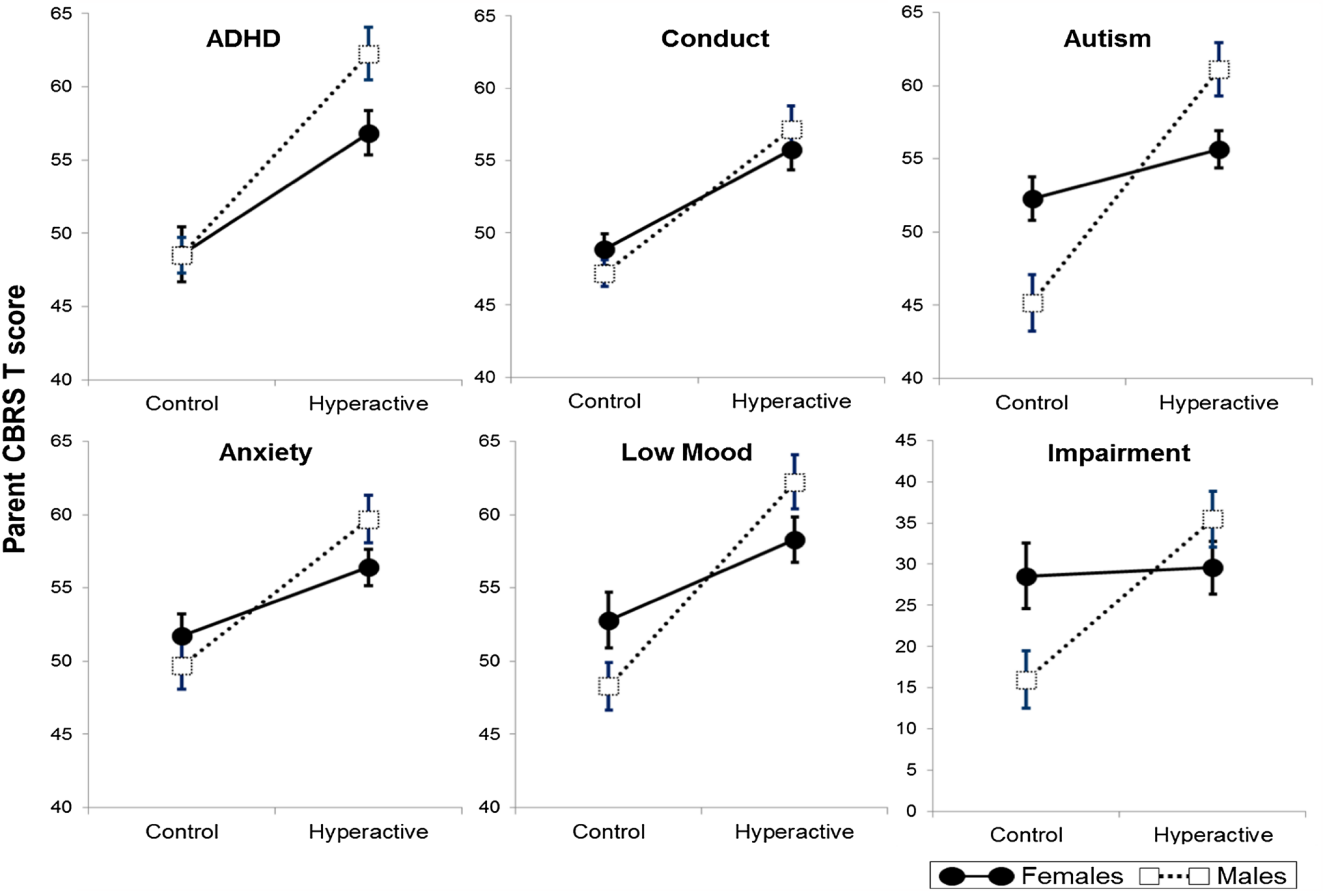
The mean scores of adolescent/young adult mental health and impairment outcomes for male versus female individuals in the hyperactive and control groups. *Error bars* = SE

Figure 1 shows the levels of adolescent/young adult ADHD, conduct, mood, anxiety, ASD symptoms, and impairment as a function of preschool hyperactivity and sex of child. There was a multivariate effect of group [*F* (1, 247) = 9.08, *p* < 0.001], no effect of sex [*F* (1, 247) = 2.00, *p* = 0.079], but the multivariate group by sex interaction was significant [*F* (1, 247) = 3.36, *p* = 0.006]. Simple main effects (hyperactive greater than controls) were present for all five outcomes for males (Table 3) and for all but ASD for females (Table 3). The size of these effects was substantially larger for males (average Cohen’s *d* = 0.97) than females (average *d* = 0.64). Univariate group by sex interactions (see Fig. 1) were significant for mood [*F* (1, 247) = 4.91, *p* = 0.028] and ASD symptoms [*F* (1, 247) = 13.12, *p* < 0.001]. They approached significance for anxiety [*F* (1, 247) = 2.98, *p* = 0.085] and ADHD [*F* (1, 247) = 2.93, *p* = 0.088]. For impairment, there was a main effect of group [*F* (1, 240) = 7.29, *p* = 0.007] and a significant group by sex interaction [*F* (1, 240) = 5.91, *p* = 0.016]. For males, the effect of group was highly significant [*F* (1, 114) = 12.58, *p* = 0.001; *d* = 0.74], but for females, it was non-significant [*F* (1, 126) = 0.04, *p* = 0.85; *d* = 0.13]. Table 4 shows the clinical significance of these effects in terms of the proportion of hyperactive and control individuals meeting the standard CBRS binary cutoffs based on the DSM-IV criteria. After correction for multiple testing, the effects of group were significant for males in all domains apart from anxiety, but for females, no effects were significant. When other preschool behavior problem scores were added as covariates to the MANOVA, the multivariate effects of group remained significant [*F* (1, 193) = 3.44, *p* = 0.005], sex remained non-significant [*F* (1, 193) = 1.25, *p* = 0.289], and the group by sex interaction also remained significant [*F* (1, 193) = 2.64, *p* = 0.025]. For males, the effects of group, although reduced in size, remained significant for all outcomes (Table 3). For females, however, the effect of group was no longer significant for any outcome (Table 3). For impairment, the effect of group [*F* (1, 189) = 2.60, *p* = 0.11] and the group by sex interaction were no longer significant [*F* (1, 189) = 2.70, *p* = 0.10]. For females, there were a number of significant associations between covariates and specific outcomes within these multivariate models. Early conduct problems were related to later ADHD [*F* (1, 102) = 8.43, *p* = 0.005], conduct [*F* (1, 102) = 9.82, *p* = 0.002], mood [*F* (1, 102) = 7.07, *p* = 0.009], and impairment [*F* (1, 98) = 4.06, *p* = 0.047] at follow-up, and early emotional problems were related to later anxiety [*F* (1, 102) = 8.58, *p* = 0.004]. For males, however, the only association, other than those involving preschool hyperactivity, was between preschool conduct problems and later impairment [*F* (1, 86) = 5.38, *p* = 0.023].

**Table 3:** Univariate outcomes of multivariate analysis of variance (MANOVA) to explore parent-rated mental health problems at young adult follow-up as a function of group and sex: with and without covariates of baseline behaviour checklist (BCL) subscales included in the model

Mental disorder	Effect of group (hyperactive vs. control) Without covariates in model				Effect of group (hyperactive vs. control) With baseline BCL covariates included in model			
	Male		Female		Male		Female	
	*F*	*P*	*F*	*P*	*F*	*P*	*F*	*P*
ADHD	26.61 (1,117)	<0.001	13.11 (1,130)	<0.001	11.57 (1,86)	0.001	0.78 (1,102)	0.379
Conduct	17.41 (1,117)	<0.001	10.27 (1,130)	0.002	7.49 (1,86)	0.008	0.68 (1,102)	0.410
Mood	23.65 (1,117)	<0.001	4.62 (1,130)	0.033	4.06 (1,86)	0.035	0.13 (1,102)	0.722
Anxiety	15.45 (1,117)	<0.001	4.77 (1,130)	0.031	6.92 (1,86)	0.01	0.83 (1,102)	0.365
ASD	30.20 (1,117)	<0.001	2.54 (1,130)	0.114	11.81 (1,86)	0.001	0.03 (1,102)	0.868

Mental disorders are based on Conners Comprehensive Behavior Rating Scales T scores. Baseline BCL measures include: conduct, emotional, sleep, toileting, and feeding problems

**Table 4:** The percentage of males and females rated as hyperactive at age 3 that go on to the meet validated thresholds on the subscales of the parent-rated mental health problems at young adult follow-up

Mental disorder	Hyperactive (%)		Control (%)		Statistics (χ^2^)		
	Male, *n* = 79	Female, *n* = 87	Male, *n* = 40	Female, *n* = 46	Full group	Male	Female
ADHD	25.3	11.5	2.5	2.2	**12.67** ^**+**^	**9.51** ^**+**^	3.45
Conduct	32.9	27.6	2.5	8.7	**19.62** ^**+**^	**14.00** ^**+**^	6.46
Mood	27.8	33.0	5.0	19.6	**9.67** ^**+**^	**8.61** ^**+**^	2.67
Anxiety	24.1	31.0	10.0	19.6	5.01	3.36	2.01
ASD	21.5	16.1	0	2.2	**15.67** ^**+**^	**10.04** ^**+**^	5.83

Mental disorders are based on Conners Comprehensive Behavior Rating Scales

^+^ Significant at *p* < 0.05 when corrected for multitests with correlated outcomes

*n*’s may differ slightly due to missing data

**Fig. 2:**
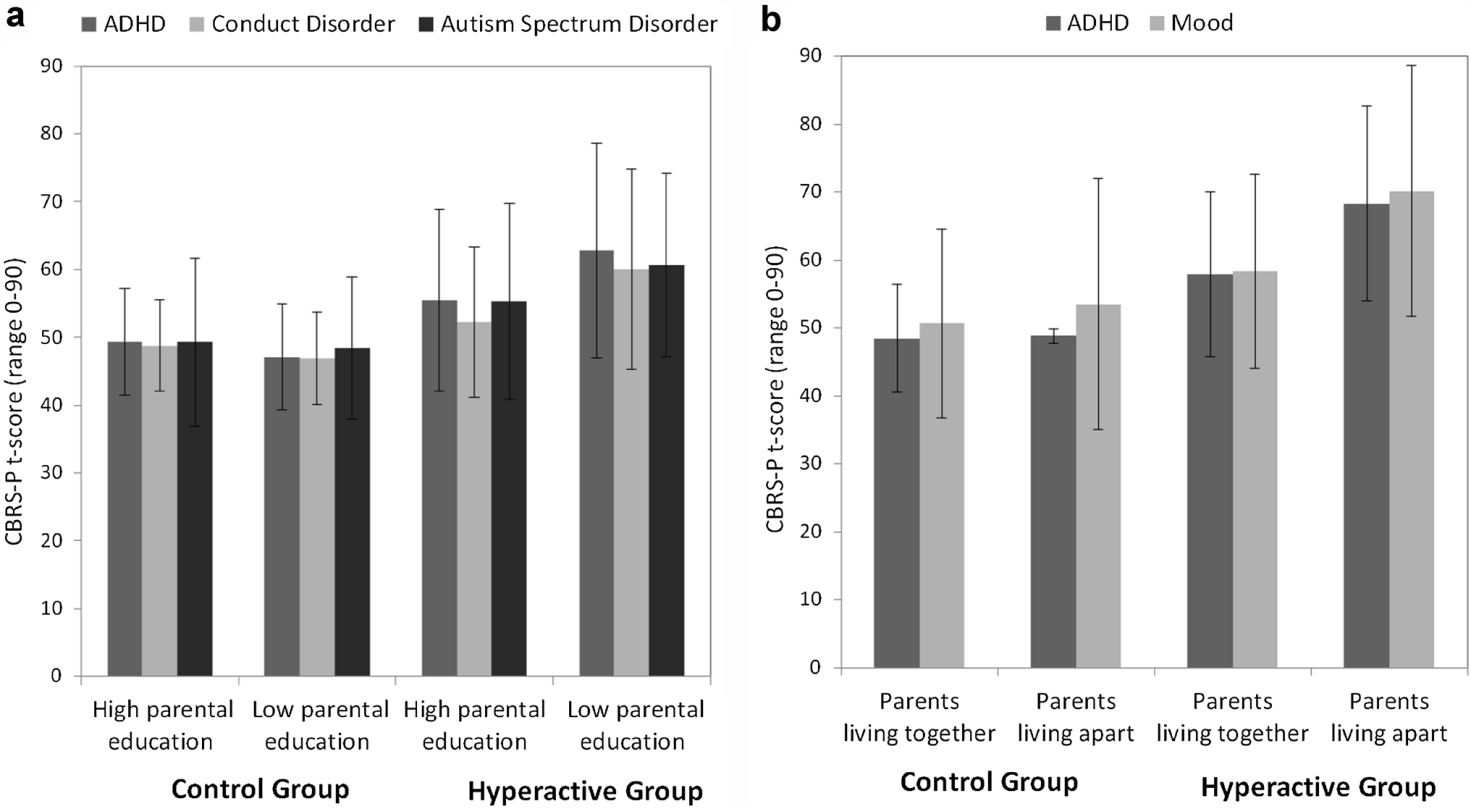
The long-term mental health effects (measured by parental reported mean Conners CBRS *t*-scores) of family factors for hyperactive versus control participants, presented by **a** parental education status (high vs. low education) and **b** parental living status (together vs. apart) when child was aged 3 years. *Error bars* represent standard deviations

When the analysis was restricted to the hyperactive group, regression models revealed no effects of preschool behavioral and developmental status or prenatal and perinatal difficulties on adolescent/young adult outcomes (see Table 5). However, significant effects were seen for a number of demographic and family background variables. Consistent with our previous analysis, male sex predicted greater ADHD, mood problems, and ASD. Moreover, parents’ living apart when the child was aged 3 years was an independent predictor of ADHD, conduct, and mood problems. Low parental education was a predictor of ADHD, conduct problems, and ASD. Following on from this analysis, we conducted a series of post hoc ANOVA with hyperactivity (hyperactive vs. control), family risk factor (present or absent) and sex to see if parental low education and parental separation moderated the long-term risks associated with early hyperactivity (i.e., effects specific to the hyperactive group) or if the effects were general across low- and high-hyperactive preschool children alike. These analyses were limited to the family factors and outcomes where a significant association had been found. Alongside the predicted effects of preschool hyperactivity (*F*s > 5.70; *p*s < 0.018) for all selected outcomes, there were significant interactions between low parental education and preschool hyperactivity for ADHD [*F*(1, 242) = 7.34, *p* = 0.007], conduct problems [*F*(1, 242) = 9.18, *p* = 0.003], and ASD [*F*(1, 242) = 4.63, *p* = 0.032]. There were main effects of parental separation for ADHD [*F*(1, 236) = 3.94, *p* = 0.048]; and mood [*F*(1, 235) = 3.92, *p* = 0.049] and a trend for conduct problems (*p* = 0.07). Figure 2a, b shows that low parental education and parental separation increased the risk of poor outcome for the hyperactive group only.

**Table 5:** Results of regression analyses within the hyperactive group (*n* = 167) to identify (1) demographic, (2) child behavioral and developmental characteristics, and (3) prenatal and perinatal risk predictors of long-term outcomes

Preschool predictors	Adolescent/Young adult outcomes					
	ADHD, *ß* (95 % CI)	Conduct, *ß* (95 % CI)	Mood, *ß* (95 % CI)	Anxiety, *ß* (95 % CI)	ASD, *ß* (95 % CI)	Impairment, *ß* (95 % CI)
Demographics						
Socio-economic deprivation	1.10 (−0.07, 2.28)	1.05 (−0.01, 2.12)	1.59 (0.35, 2.82)	1.28 (0.21, 2.35)	0.65 (−0.50, 1.81)	0.04 (−0.01, 0.08)
Parents’ living apart	**10.24** ^**+**^ **(3.93, 16.54)**	**9.55** ^**+**^ **(3.81, 15.28)**	**11.19** ^**+**^ **(4.54, 17.84)**	4.39 (−1.35, 10.12)	2.45 (−3.76, 8.67)	0.23 (0.01, 0.48)
Low parental education	**6.60** ^**+**^ **(2.23, 10.97)**	**7.06** ^**+**^ **(3.09, 11.04)**	1.25 (−3.36, 5.86)	−1.32 (−5.30, 2.66)	**5.00** ^+^ **(0.70, 9.31)**	0.05 (−0.13, 0.22)
Sex	**6.02** ^**+**^ **(1.69, 10.34)**	2.03 (−1.90, 5.97)	**4.67** ^**+**^ **(0.11, 9.23)**	3.65 (−0.28, 7.59)	**5.65** ^+^ **(1.39, 9.91)**	0.14 (−0.03, 0.30)
Behavior problems						
Hyperactivity	0.21 (−0.14, 0.55)	0.10 (−0.22, 0.42)	0.05 (−0.31, 0.40)	0.09 (−0.21, 0.39)	−0.19 (−0.52, 0.14)	0.00 (−0.01, 0.01)
Conduct	0.17 (−1.61, 1.95)	0.95 (−0.69, 2.58)	1.77 (−0.05, 3.59)	0.13 (−1.40, 1.67)	0.50 (−1.18, 2.18)	−0.00 (−0.07, 0.06)
Emotional	1.06 (−1.26, 3.38)	0.69 (−1.44, 2.82)	0.39 (−1.98, 2.77)	1.86 (−0.14, 3.86)	0.82 (−1.37, 3.01)	0.06 (−0.03, 0.14)
Sleep	1.18 (−0.16, 2.52)	0.58 (−0.65, 1.82)	0.91 (−0.46, 2.28)	0.47 (−0.68, 1.63)	0.25 (−1.02, 1.51)	0.02 (−0.04, 0.06)
Toileting	1.24 (−0.49, 2.96)	0.35 (−1.24, 1.93)	0.83 (−0.94, 2.59)	1.08 (−0.40, 2.57)	1.96 (0.33, 3.58)	0.05 (−0.01, 0.12)
Feeding	0.02 (−1.90, 1.93)	0.28 (−1.48, 2.04)	1.15 (−0.81, 3.10)	−0.90 (−2.54, 0.75)	−0.10 (−1.91, 1.70)	−0.02 (−0.09, 0.05)
Developmental delay						
Speech	3.45 (−2.78, 9.69)	4.93 (−0.81, 10.65)	3.99 (−2.39, 10.37)	3.31 (−2.06, 8.69)	5.49 (−0.39, 11.37)	0.07 (−0.16, 0.31)
Cognition	−0.18 (−9.49, 9.13)	−5.59 (−14.15, 2.96)	−6.35 (−15.88, 3.17)	−2.38 (−10.40, 5.65)	−4.60 (−13.39, 4.18)	−0.29 (−0.63, 0.06)
Prenatal and perinatal risk factors						
Premature	−4.29 (−16.28, 7.71)	0.18 (−10.85, 11.21)	−5.83 (−18.18, 6.52)	−3.99 (−14.35, 6.38)	−1.83 (−13.18, 9.53)	−0.17 (−0.60, 0.27)
Low birth-weight	6.61 (−3.53, 16.74)	0.66 (−8.67, 9.98)	6.85 (−3.59, 17.29)	2.14 (−6.62, 10.91)	4.04 (−5.56, 13.64)	0.16 (−0.22, 0.53)
Birth complications	4.19 (−1.49, 9.87)	0.84 (−4.38, 6.06)	4.36 (−1.49, 10.21)	2.59 (−2.32, 7.50)	1.08 (−4.29, 6.46)	0.18 (−0.03, 0.39)

All measures of mental disorder categories at adolescent/young adult outcome are derived from the Conners Comprehensive Behavior Rating Scales. ADHD relates to inattentive type and//or ADHD hyperactive Impulsive type, conduct relates to conduct disorder and/or oppositional defiant disorder, mood to major depressive and/or manic episode, anxiety to general anxiety disorder and/or social phobia and/or obsessive compulsive disorder. Impairment is derived from Weiss Functional Impairment Rating Scale; preschool hyperactivity is based on the Werry–Weiss–Peters Activity Rating Scale. Preschool behavior problems are based on the behavior checklist. Deprivation is based on the Carstairs index. Sidak’s alpha (*p* < 0.05) level corrected for 6 tests with a multicorrelation of 0.63 = *p* < 0.009)

^+^ Significant at *p* < 0.05 when corrected for multiple tests with correlated measures

## Discussion

In order to target early interventions for childhood problems, so as to improve later life outcomes, it is important to be able to identify early risk markers. In the past, hyperactivity—as a precursor to ADHD—has been identified as a possible precursor of later mental health problems and, therefore, a potential target for early intervention [5, 15–17]. The process of early prediction of later problems is complicated by the existence of developmental discontinuities in trajectories from preschool hyperactivity—many preschoolers showing hyperactivity do not go on to develop clinically significant problems [38]. In the current paper, we attempted to identify early markers of later problems that might index risk more precisely. This large-scale longitudinal study was planned to identify which preschool children go on to develop ADHD and other behavioral problems later in life. As far as we are aware, this is the largest study conducted with the specific focus on high-risk preschoolers. There were a number of findings of note.

First, at baseline, preschool hyperactivity was not significantly associated with sociodemographic and family related factors. The hyperactive group had more problems than controls across all measured behavior problem domains—providing further support for the association between hyperactivity and co-occurring emotional, behavioral, and developmental problems more generally in the preschool period [11]. Consistent with the literature, associations with hyperactivity were particularly marked for conduct and sleep problems [62, 63]. This overlap between clinical problems is consistent with previous findings in preschool children [64] and older children with ADHD [65–67] and highlights the need to consider other problems when assessing the long-term power of hyperactivity to predict poor outcomes over time.

Second, there were no sex differences at baseline, either in the severity of hyperactivity, or in the levels of co-occurring problems. Male and female individuals in the hyperactive group were equally affected. Levels of preschool hyperactivity in Table 1 for the selected control and hyperactive samples as a whole suggest that this was not an artifact of bias due to sampling or attrition, but reflected a more general equivalence of hyperactivity and associated problem levels in males and females in the preschool period. This is consistent with previous findings of similar, if not higher, symptom severity in hyperactive girls versus hyperactive boys, at least during early childhood [16].

Third, at the group level, there were significant continuities between early hyperactivity and later problems (Table 3). There were homotypic continuities linking preschool hyperactivity to later ADHD and heterotypic continuities linking hyperactivity to conduct problems, mood, and ASD. This was particularly striking given (1) the length of follow-up in the current study and (2) that the criteria for inclusion in the preschool hyperactivity group were quite lax (top 17.2 % of the sample population). A number of prior studies have established preschool hyperactivity as a risk factor for the development of ODD and CD and criminal activity more generally over the long term [11, 13, 16, 45, 68]. The effects on ASD outcomes are more novel. However, a study exploring pragmatic language difficulties in children age 4 years, established a relationship between pragmatic language impairment, activity levels, and externalizing behaviors [69]. This relationship was suggested by the authors as a potential early marker of underlying ADHD and/or autism [69]. It is possible that given the non-specific nature of the baseline measure of hyperactivity used in this study, high levels of activity were marking both the neurodevelopmental risk associated with nascent ASD traits and the behavioral risk for early emerging externalizing problems. In the future, it will be interesting to try to identify whether more specific markers of activity style can be identified that predispose individuals to either an externalizing problems pathway or an ASD pathway.

Fourth, despite their similarity at baseline, the power of preschool hyperactivity to predict long-term outcomes was rather different for males and females. Two points are worthy of further note. 1) when continuous outcomes were considered, the size of the association between preschool hyperactivity was about 30 % greater for males compared with females (evidenced by a significant sex by group interaction) with this sex difference seen across both internalizing and externalizing disorders (Fig. 1). This is consistent with previous findings of a greater likelihood of externalizing problems in males with ADHD [21, 24–26], but inconsistent with a greater likelihood of internalizing problems in females with ADHD [21, 22, 25, 26, 30]. 2) when binary outcomes were considered, hyperactive female preschoolers were not significantly more likely than controls to meet standard cutoffs on the CBRS (Table 4). The difference between the sex effects in the analyses of continuous and binary outcomes is likely due to reduced power typically found when continuous variables are categorized. One factor that may have mitigated against finding significant effects for females was that, at follow-up, levels of disorder were higher in the control group for females than males (Table 4). In this regard, it is noteworthy that nearly 20 % of control females met the cutoffs for depression and anxiety.

Fifth, the long-term negative outcomes seen in males appeared to be driven almost entirely by preschool hyperactivity rather than other behavioral problems. Prior studies have suggested that other types of internalizing and externalizing problems are also important predictors and may even account for the majority of poor outcomes. For instance, the existence of conduct problems [16, 39], emotional difficulties [39], sleep problems [70], and DD [39, 64] have all been shown to be an important factor predisposing hyperactive children to poor outcome. For females, preschool conduct and emotional problems rather than hyperactivity were the main driver of poor outcomes. The reason that hyperactivity did not emerge as a significant marker for the high prevalence of problems in adolescence/early adulthood in females is unclear but may constitute clinically important findings. Of note, recent studies investigating infant markers in autism have also reported sex differences [71, 72]. Further exploration is needed to interpret these observed sex differences in terms of either genetic or maturational mechanisms, or proximal risk [36, 71, 72]. As discussed, there is a growing evidence of different developmental and pathological trajectories for males and females with ADHD [4, 21, 23, 27, 30, 33, 34], so preschool hyperactivity may not be the most predictive feature of later mental health problems in females with ADHD. For example, inattentiveness has been identified as a more prominent feature of ADHD in females than in males [21, 26, 33, 34]. Although there was no preschool inattention measure available at the time, preschool inattention may have been a better predictor of female mental health outcomes. In a prevalence study of ADHD by subtype and gender, Willcutt found that the sex ratio for the ADHD subtype of inattentiveness was equal for males and females during the preschool period [29]. Females with inattentive ADHD have been shown to fare worse over time than males with inattentive ADHD, particularly in terms of social functioning and internalizing symptoms [22]. Alternatively, the parent perception of observed behavior in males and females may differ. Vukojevic et al. established that parents recognized early symptoms of ADHD more frequently in males, whereas teachers recognized them more frequently in females [73]. The authors explained this in terms of teachers being more readily able to make peer group comparisons [73]. It is yet unknown whether this trend in parental versus teacher gender perception bias is a more general phenomenon; whether it is a feature of externalizing versus internalizing symptoms; and whether it extends to older children and adolescents.

Sixth, few factors helped to identify which preschoolers with hyperactivity are at specific risk for poor outcomes. Interestingly, neither severity of hyperactivity symptoms nor problems in other domains were important. In fact, what marked out those with extra risk were family factors—low parental educational level (no qualifications above those taken in school at age 16 years) and parents living apart at the time of the preschool assessment both significantly increased the long-term risk of poor outcomes across multiple domains. Previous studies have linked these factors to poor outcomes. For instance, low maternal education (defined as ≤9 years schooling) was associated with an increased risk of continued aggressive behaviors in a cohort of children displaying aggressive behaviors in preschool [74]. Low parental education was also a significant indicator of ADHD in a very large cohort study to assess potential causal pathways of ADHD [46]. Family breakdown has also been associated with negative behavioral outcomes. For example, stressful life events (including parental divorce) was shown to be a predictive factor of later ADHD diagnosis in a study of hyperactive preschoolers [42] and family conflict mediated the relationship between ADHD and socioeconomic disadvantage [46]. In the current study, low parental education and family breakdown seemed to increase risk only in the hyperactive group. Interestingly, these factors operated independently of each other and were not due to general patterns of social deprivation.

From a clinical perspective, the results of the current study raise a number of issues. First, early screening for hyperactivity in the preschool period may facilitate the cost-effective targeting of early intervention efforts to reduce long-term burden for mental health problems—specifically in males. Clearly, any recommendations in this regard are tempered by the availability of effective preventative approaches. Importantly, the value of early screeners for hyperactivity extended from the ADHD domain to externalizing and internalizing problems as well as ASD. Second, although early hyperactivity is an independent predictor of a range of negative outcomes—this does not mean that early interventions should necessarily focus on reducing hyperactivity per se—hyperactivity in preschoolers may have different underpinnings to hyperactivity related to ADHD in the long term. Our results suggest that preschool hyperactivity may be a marker of other underlying deficits that underpin the poor outcomes. So, for instance, while early hyperactivity may indicate a raised level of risk for ASD, treating hyperactivity is unlikely to reduce that risk but could result in an earlier identification and appropriate intervention for that condition. Third, the results suggest that conduct and emotional problems (rather than hyperactivity) may be important markers of long-term risk in females. Exploring the early risk markers of poor mental health in males versus females in terms of genetic/phenotypic protective/vulnerability factors is a major health priority. Finally, hyperactive children in underachieving and dysfunctional households may be at risk, and this could provide an important focus for targeted resources.

The study had a number of limitations to take into account. First, attrition was high, although our analysis suggests that the results are unlikely to be the result of biases due to this. Baseline characteristics were similar for responders and non-responders. Second, baseline measures were based only on parental report. Corroborating these reports with other informants would have strengthened the reliability of the baseline data. Third, as noted above, we did not have a measure of preschool inattention. Given the evidence of the association with inattentiveness and negative long-term outcomes for females [22], preschool inattentiveness may have been an important marker for mental health outcomes in females even in the absence of the effects of hyperactivity. Fourth, we did not include a measure of parental mental health at baseline, and therefore, we cannot rule out that this variable was driving some of the family factor effects. For example, depression in mothers can lead to decreased warmth toward the child [75, 76] but also adult ADHD is known to have a negative effect on parenting [76–80]. The effects of family factors may, therefore, not only reflect environmental risk, but also be a proxy of genetic risk. The presence of adult ADHD, for instance, would be a marker of the genetic transmission of the condition; increasing the likelihood of the long-term presence of ADHD within the child. Although cognitive delay was measured at baseline, there was also no measure of IQ at follow-up, which may have been a confounding factor. As the control group was smaller than the hyperactive group, this may have reduced our power to detect clinically important effects, especially given the need to control for multiple testing. Last, but not least, mental health at follow-up was based on parent-rated symptoms, which is not the same as meeting criteria for a mental health disorder. As the mental health measure in this study relied on parental awareness, parents may have been more aware of later behavioral problems in males than emotional problems in females.

In summary, this large-scale longitudinal study is the first of its kind with the power to identify which high-risk factors in the preschool children predict later development of ADHD and associated mental health and behavioral problems. We provide evidence for the singular importance of preschool hyperactivity as a marker of long-term risk for mental health in males. Future studies should incorporate additional ways of identifying hyperactivity and other potential preschool markers in males as well as females, perhaps by including a measure of preschool inattentiveness, observations from other informants, and including measures of potential moderating or protective factors that may have a differing impact between the sexes over time. Focusing on understanding the different pathways from preschool hyperactivity as a general marker for outcomes as diverse as ADHD and ASD may better characterize the preschool mental health risk profile for females.
